# When the impossible becomes possible: COVID-19’s impact on work and travel patterns in Swedish public agencies

**DOI:** 10.1186/s12544-021-00471-9

**Published:** 2021-02-20

**Authors:** Lena Winslott Hiselius, Peter Arnfalk

**Affiliations:** 1 Department of Technology and Society, Lund University, Box 118, 221 00 Lund, Sweden; 2 K2—The Swedish Knowledge Centre for Public Transport, Bruksgatan 8, 222 36 Lund, Sweden; 3 The International Institute for Industrial Environmental Economics, Lund University, 221 00 Lund, Sweden

**Keywords:** COVID-19, Telework, Virtual meetings, Travel restrictions, Transport sustainability

## Abstract

**Background:**

The COVID-19 pandemic has rapidly led to some of the most revolutionary changes in private and professional life around the world. While the extent and duration of these changes are not certain, they have already had a great impact on travel patterns. This is also the case in Sweden, despite its relatively liberal approach to restrictions, which relies on voluntary measures such as social distancing and self-monitoring for symptoms.

**Methodology:**

Due to the pandemic, a shift to telework and virtual meetings is being tested in what can be seen as a large-scale experiment, and the knowledge and experience from that experiment may have lasting effects on everyday life. This study seeks to analyse the effects of government and public agencies’ recommendations on meeting and travel behaviour on employees at five public agencies in Sweden.

**Results:**

The results indicate that the public authorities surveyed were well prepared and had a ‘backup collaboration solution’, at least technically, to make a rapid behavioural shift when travel was not an option. Though the Swedish government’s and Public Health Authority’s strong recommendations have led to the most dramatic reductions in work-related travel in modern times, the operations in Swedish agencies continue to function, along with the employees’ communications and collaborations. These results indicate that there is great potential for digital tools to influence if and how we commute and make business trips. The COVID-19 pandemic has shown that such tools can make the impossible possible.

## Introduction

The COVID-19 pandemic has rapidly led to some of the most revolutionary changes in private and professional life around the world. Self-isolation and travel restrictions have resulted in a dramatic reduction in the demand for passenger transport, including public transport, as potential passengers are concerned about being infected by other travellers. While the extent and duration of these changes are not certain, they have already had a great impact on travel patterns. Aloi et al. [2] analysed the effect that the quarantine measures imposed in Spain on 15 March 2020 had on urban mobility in the northern city of Santander and the analysis revealed an overall mobility fall of 76%. Public transport use decreased by up to 93%. According to a report from the McKinsey Centre for Future Mobility [27], public transit ridership has fallen by 70 to 90% in major cities across the world. Other studies reporting large changes in traffic flow and modal share (especially reduction in public transport ridership) are Bucsky [9], Saladié et al. [30] and Tan and Ma [36].

Travel patterns have been affected also in Sweden, despite the country’s relatively liberal approach to restrictions, relying on voluntary measures such as self-imposed social distancing, self-monitoring for symptoms, staying home when ill, and practicing good hand hygiene. Swedes are advised to avoid unnecessary private and business travel. Regarding commuter travel, the advice from the public health agency is to work from home when possible and permitted by the employer to do so, maintain physical distance in public transport, and avoid travelling at rush hour unless necessary. In Jenelius and Cebecauer [24] analysing the effects due to the measures towards COVID-19 in Sweden shows a severe decrease in public transport ridership (40%–60% across regions in Sweden) compared with other transport modes. Further, analyses by Almlöf et al. [1] show that education level, income, age as well as workplace type are strong predictors, of the propensity to stop travelling by public transport in Stockholm, the capital of Sweden.

In addition to helping prevent the spread of COVID-19, the transport system is under pressure to reduce its greenhouse gas emissions. Transport is responsible for almost 25% of global energy-related greenhouse gas emissions, a share that has been shown to be increasing [21]. The promotion of more sustainable and energy-efficient travel behaviour is of considerable interest, and there is broad agreement among transport researchers that the level of traffic must be reduced in order for the sector to contribute to more sustainable development [16, 41].

Due to the COVID-19 pandemic, there has been an increase in the use of telework and virtual meetings and a great reduction in commuting and business trips. With the help of various technical solutions such as telephones, computers, tablets, smartphones and special audio-, web-, and videoconferencing equipment, workers are meeting and collaborating virtually in real time. This is, of course, nothing new: the number of organisations using virtual meetings and flexible working places was already growing steadily before the pandemic [19]. However, despite investments in IT infrastructure and equipment and the potential benefits of virtual collaboration [40], many organisations have been failing to convince the employees to make full use of these solutions (e.g. [4, 13]). Business trips in particularly have been regarded as a part of business culture that is difficult to replace [22].

Also due to the pandemic, a shift to telework and virtual meetings is being tested in what can be seen as a large-scale experiment, and the knowledge and experience from that experiment may have lasting effects on everyday life. The paper analyses its implications for the meeting and travel behaviour of employees at five public agencies in Sweden. The studied agencies^1^ are since 2011 part of the REMM project (Virtual Meetings in Public Agencies), a project aiming to increase and improve the use of virtual meetings in Swedish public agencies (Arnfalk et al, [5]). Based on these agencies’ involvement in REMM they may be judged as being well equipped regarding virtual meetings when the government and public health authority’s recommendations took effect. This study focuses on analysing the effects of these circumstances on meeting and travel behaviour though Sweden’s relatively liberal use of restrictions, and the employees’ perceptions of these changes.

## Background

### Virtual meetings and teleworking

Most public and private organisations today rely on communication and interaction between people working in geographically dispersed locations. As a result, business travel has increased significantly [23]. With travel costs rising and employees spending more of their time on planes and in cars, companies are seeking alternatives. One option is virtual meetings, which are being used not only to reduce business travel but also to make business operations more efficient in general. A *virtual meeting* is real-time communication between geographically separated parties by means of digital devices [40]. Such a non-travel alternative may be considered before a business trip is booked. The main advantages of virtual meetings for companies are streamlined operations, decreased travel costs, time savings, and reduced environmental impact. Another possible benefit is increased opportunities for national and international cooperation and increased competitiveness. For the individual, virtual meetings mean less time spent on business trips, which may have positive effects on both work and leisure [35].

Virtual meetings require the purchase of equipment and the provision of support, as well as also a new meeting culture. For virtual meetings to have an impact, it is important for the company to provide training, information, support, and guidelines for how and when it is to be used [14].

Most companies and other organisations that invest in virtual meetings expect them to replace travel, at least to some extent. While some individual organisations have reduced their per-capita business travel costs up to 70% through increased use of virtual meetings [37, 38], travel reduction figures in the range of 20–35% are more commonly reported [3]. The substitution potential of virtual meetings has long been a subject of debate, and many estimation attempts, such as Buttazzoni et al. [10], have predicted that virtual meeting would replace 25–65% of business travel in 2030 and 33–90% in 2050. The Climate Group [15] has estimated that approximately 30% of business travel could be replaced by videoconference by 2020.

With teleworking, employees are given the opportunity to perform some or all of their duties at home or at an alternative location [8, 11, 17], an approach to work that is increasingly being adopted in workplaces such as public organisations [11, 18]. The advantages often cited in relation to telework include increased freedom and flexibility and reduced need for travel, resulting in lower emissions and travel costs [6]. However, the positive environmental effects are limited if workers’ free time is used for other trips [32]. Elldér [20] analysed the links between telework and daily travel activities in Sweden. His results show that those who telework full days make significantly fewer and shorter trips and are also more likely to only walk or cycle for transport than those who do not telework. Company benefits of telework include primarily a reduction in cost of office space and more motivated employees, which can contribute to increased productivity and reduced staff turnover [6].

### Workplace travel plan/policy

The management of business trips refers not only to the behaviour and conditions of the individual traveller, but also to guidelines and the organisational culture surrounding business trips [7, 12, 25]. Business travel is usually regulated in a company travel policy that contains regulations on aspects such as how to travel, what means of transport and what suppliers to use, what degree of comfort is allowed (e.g., economy or business class), and what kind of ticket to use. On the other hand, even when there is a travel policy, research has shown that employees, especially managers, have a relatively large degree of freedom in deciding whether and in which travel mode to undertake a trip [26].

Travel policies often include guidelines regarding virtual meetings, such as through audio-, web-, and videoconferencing [28]. Various initiatives have been launched in order to increase the use of virtual meetings. One example is the REMM project (Virtual Meetings in Public Agencies, remm.se) initiated by the Swedish Government in 2011. This is an active investment in increasing the proportion of virtual meetings in 19 government agencies in Sweden resulted in an average reduction in CO_2_ emissions from business travellers per employee by 25% over a seven-year period, which can be compared to other Swedish authorities, where corresponding emissions decreased by 6% during the same period [33]. By actively working to integrate virtual collaboration as a natural and preferred part of their meeting culture, the public agencies have managed to gradually change the attitudes and habits surrounding business travel. However, during recent years, the trend of reduced CO_2_ emissions from business travel has levelled out [34]. Surveys conducted in the REMM agencies before the COVID-19 pandemic showed that nearly half (45%) of the employees travelling for business believed that none of their business trips could be replaced with virtual meetings, while only 15% on average thought that more than half of their business trips could be replaced.^2^

## Method

The participants in this study were employees at five public agencies in Sweden. The selected agencies are involved in the REMM project and engaged in improving and increasing their virtual meetings. Due to this engagement, the organisations have invested in the necessary technical infrastructure and equipment, and the knowledge level among the staff in how to collaborate virtually is relatively high [5]. Selected to represent various areas within Swedish state administration, the agencies are: the Transport Agency, the Environmental Protection Agency, the Energy Agency, the Agency for Digital Government, and the Public Employment Service.

The selected agencies (Table 1) represent the general location pattern of all agencies in Sweden, with a mixture of agencies located in major cities (> 300,000 inhabitants) and in cities with approximately 100,000 inhabitants.

**Table 1:** Statistics on Selected Public Agencies

Organisation	Number of employees	% Women	% Men	Main office
Transport Administration	9088	40%	60%	Borlänge
Environmental Protection Agency	620	65%	35%	Stockholm & Östersund
Energy Agency	444	61%	39%	Eskilstuna
Agency for Digital Government	60	59%	41%	Stockholm & Sundsvall
Public Employment Service	9665	65%	35%	Stockholm

Source: Annual Reports of 2019 and [31]

To capture the effects of telework and virtual meetings, the study focused mainly on office workers, randomly selected by contact persons at each of the agencies. In the design of the study, some priority was given to a quick start-up with the aim to capture the immediate effects of the COVID-19 pandemic. The targeted group was civil servants (office workers) and only a small share (three respondents) commented in the survey that they were not able to telework due to special technical/practical requirements.

For the survey, a questionnaire was distributed via email to a number of selected employees. The survey contained questions on demographic factors, commuting and business travel, and attitude toward and choice of travel modes, as well as questions related to the use of telework and virtual collaboration. To enable analyses of changes in behaviour and attitudes, the questionnaire contained questions relating to these aspects before and during the time of the survey (i.e. before and during the COVID-19 pandemic).

It is known that self-report surveys are prone to various types of response bias, including social desirability bias, acquiescence bias, and satisficing, which might undermine the validity (e.g. [29]). This study attempted to limit the occurrence of biases by using different types of response alternatives (scale, multiple choice, and open questions).

The web survey was conducted from mid-April until the beginning of May 2020, using the survey tool Survey Monkey. In Sweden, the spread of COVID-19 peaked during this period, with many people being hospitalised and an intense media coverage. The Swedish population was strongly advised to work from home if possible, including employees at the studied agencies. At this time, an email with a link to the questionnaire was sent out by the contact person at the agency. The respondents were assured anonymity, and no identifying information was connected to each respondent. The survey email was sent to 60–360 employees per agency (1020 in total), and a total of 719 answers were received (a response rate of 70%). The high response rate may be attributed to the fact that the study was launched at a relatively early stage of the COVID-19 pandemic (and before survey fatigue had escalated), but also the way the survey was distributed: an email was sent by a management representative in each organisation, requesting the employee to answer the survey.

## Results

### Background statistics

The background statistics of the respondents are presented in Table 2. There are relatively few respondents from the youngest and oldest age groups, with a majority representing 30–39-year age group. The gender balance varies among the agencies. Generally, more females than males answered the questionnaire, except in the Public Employment Service, where the majority of respondents are men. Since the distribution of the questionnaire was conducted through a contact person at each agency, there is no information regarding the age and gender of the non-respondents who received the questionnaire and thus no information on how representative this sample is for office workers at the agencies. However, in comparing the gender distribution of our sample with that of each agency based on their annual reports (presented in Table 1), there is a general congruence. (There is, however, an overrepresentation of women in the sample of the Transport Administration and an overrepresentation of men in the sample of the Public Employment Service.) In the case of the Transport Administration, the higher share of women in our survey is likely due to women being overrepresented among the office workers (versus other roles) in that agency. The difference in gender share for the Public Employment Service is likely due to the gender balance at the division that received the questionnaire.

**Table 2:** Background Statistics of Respondents Per Agency and Total

Characteristics	Share per agency					Share in all agencies
	Transport Adm.	Environmental Protection Agency	Energy Agency	Agency for Digital Government	Public Employment Service	
Age						
20–29 years	8.5%	5.6%	3.5%	3.3%	5.8%	8.5%
30–39 years	24.3%	20.4%	22.9%	16.7%	14.6%	24.3%
40–49 years	29.3%	29.6%	31.9%	43.3%	34.0%	29.3%
50–59 years	25.6%	26.9%	31.9%	26.7%	31.1%	25.6%
60–69 years	12.3%	17.6%	9.7%	10.0%	14.6%	12.3%
Gender						
Female	52.7%	64.8%	69.4%	60.0%	41.7%	56.7%
Male	47.3%	35.2%	30.6%	40.0%	58.3%	43.3%
Number of responses	317	108	144	30	103	702

### Commuting – telework

In Table 3, the responses to the question regarding the pandemic’s effects on the general work situation are presented. Based on the responses, it is clear that the pandemic has greatly affected the work situation in Sweden though the country (compared to many other countries) appealed to voluntary behavioural adjustments. Only a few respondents state that they are unaffected.

**Table 3:** Responses to the Question ‘Has COVID-19 Affected Your Work Situation?’

Organisation	Yes, very much	Yes, somewhat	No
Transport Administration	43%	51%	6%
Environmental Protection Agency	74%	24%	2%
Energy Agency	59%	38%	3%
Agency for Digital Government	68%	32%	0%
Public Employment Service	64%	33%	3%
All agencies	55%	41%	4%

*Note*: Separated by organisation and for all agencies

**Fig. 1:**
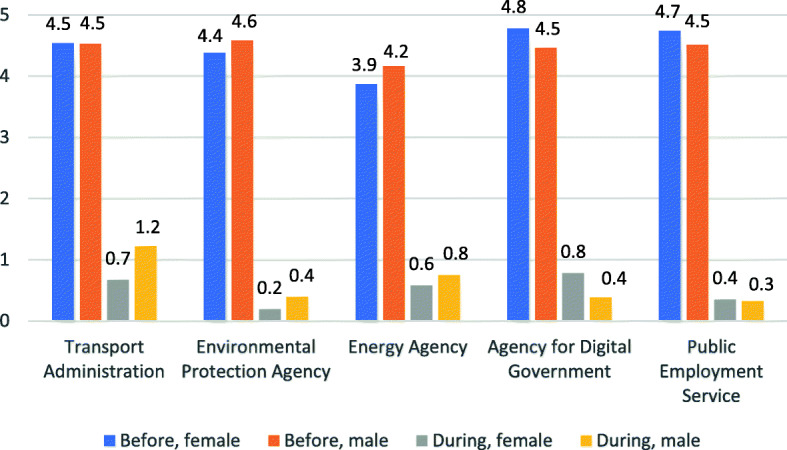
Average commuting days per week. Responses to the Question ‘How Many Days Per Week Did You Travel to Work Before, and How Many Days Do You Travel Now, During the COVID-19 Pandemic?’. *Note*: Separated by organisation and gender

The responses regarding travel to work indicate that, though liberal use of restrictions, the appeals expressed had a major impact on whether the studied office workers commuted to work. Figure 1 shows the average number of days per week the respondents commuted to work before and during the time when the study was conducted. The average number of commuting days per week dropped from 4.4 to 0.5 for women and from 4.5 to 0.8 for men. Further analyses show that of those commuting 5 days a week prior to the pandemic, 66% did not commute at all, while 16% reduced their commuting to only 1–2 days a week. These results can be interpreted as a massive increase in the number of persons working from home or another location outside of the workplace. At the same time and for both genders, there is a statistically significant difference between the agencies in the number of commuting days during the pandemic. As Fig. 1 indicates, this variation corresponds well to the variation in number of commuting days prior to the pandemic, i.e. the commuting pattern seems to have remained though on a very low level during the pandemic.

The variation in number of commuting days before the COVID-19 pandemic may be explained by the share of respondents working part-time. No question relating to working hours was included in the questionnaire, but the co-variation between number of commuting days and the share of women and respondents in ages of 20–39 years, see Table 2, indicates that this could be the case as these factors correlate with the propensity of working part time, [31].

In the survey, the respondents were asked to rate (scale 1–100) how well they thought teleworking works during the COVID-19 pandemic. In Tables 4, 5, 6, the responses are separated by organisation, gender and age, and the number of days per week commuting before the pandemic. Teleworking seems work reasonably well, with an average rating of 74. Respondents from the Public Employment Service and the Environmental Protection Agency were the most pleased with how teleworking worked. These agencies had also the largest reduction in the number of commuting days per week compared to the situation before the COVID-19 outbreak (and, correspondingly, highest increase in the level of telework).

**Table 4:** Responses to the Question ‘How Well Do You think Teleworking Works for You Now, During COVID-19” (Rating 1–100)?’

Organisation	How well telework works	
	Mean	Stdv
Transport Administration	75.06	21.65
Environmental Protection Agency	72.27	21.09
Energy Agency	71.19	20.31
Agency for Digital Government	73.41	16.96
Public Employment Service	78.47	16.74
All agencies	74.26	20.49
F-test (*p*-value)	2.17 (0.071)	

*Note*: Separated by organisation and for all agencies

**Table 5:** Responses to the Question ‘How Well Do You think Teleworking Works for You Now, During COVID-19” (Rating 1–100)?’

Commuting days per week before COVID-19	How well telework works					
	Female			Male		
	Mean	Stdv	***N***	Mean	Stdv	***N***
0	64.50	36.06	2	–	–	0
1	92.33	1.53	3	71.33	27.90	6
2	84.90	20.36	10	76.73	24.78	11
3	80.54	13.47	35	81.88	18.12	17
4	78.89	17.35	109	74.59	19.41	66
5	73.42	20.77	221	69.82	22.13	186
F-test (*p*-value)	1.14 (0.221)			1.71 (0.002)		

*Note*: Separated by number of commuting days before COVID-19

**Table 6:** Responses to the Question ‘How Well Do You think Teleworking Works for You Now, During COVID-19” (Rating 1–100)?’

Characteristics		How well telework works			
		Female		Male	
		Mean	Stdv	Mean	Stdv
Age	20–29 years	71.42	23.27	60.68	21.89
	30–39 years	73.74	20.44	71.53	23.46
	40–49 years	75.79	19.20	71.75	20.65
	50–59 years	79.03	17.74	74.44	20.83
	60–69 years	75.82	20.45	74.72	20.47
	Average	76.10	19.46	72.07	21.64
F-test (*p*-value)		1.18 (0.169)		1.47 (0.018)	

*Note*: Separated by age and gender

These results also reveal that the respondents who previously commuted 2–3 days a week thought that working from home worked most well. This may be because those who teleworked a few days a week before COVID-19 likely already have the technical and practical setup ready at home, but it could also be an effect of this survey’s inclusion of part-time workers, whose number of commuting days did not change extensively. The results also show that the more days the respondent commuted prior to the pandemic, the lower he or she ranked how well teleworking worked during COVID19.

Generally, women in the data set were more pleased with teleworking than men. Further, how well teleworking is considered to work appears to increase with age. This may be due to factors connected to the situation at work but also at home, e.g. younger employees more often need support and guidance from more senior colleagues and engage more often socially with their co-workers, both of which are more difficult to do when working from home. But younger people are more likely live in smaller homes and to have children (and disturbances) at home. Another possible explanation relates to COVID-19 in that persons in higher age groups are more concerned about contracting COVID-19 since they risk more severe health outcomes.

The respondents were also asked to reflect on the adjustment to working from home. Many expressed that there was not much of a difference: e.g. ‘I work from home but do overall the same job, albeit in a different way’ and ‘I have a good workspace with all the equipment I need, so it works well’. On the other hand, many others commented on the need for technical and physical support in order to cope with the new situation: e.g. ‘I had to furnish part of a room in the apartment as a place to work’ and ‘Among other things, I needed to buy a height-adjustable desk in order to work at home without getting (serious) trouble with my neck’. Some even expressed concerns regarding the quality of the work situation and the work carried out: e.g. ‘More work at home with poorer technical equipment (especially number of screens) and more disruptive moments (children)’ and ‘It is difficult to delegate and collaborate effectively via Skype. We are recommended to work at home as much of the time as possible. Assignments and projects require physical presence to be done well.’ Though most of the respondents worked at home all week, the results in Fig. 1 indicate that some respondents spent 1–2 days a week at the office, and their worries were captured as well: e.g. ‘We sit in an activity-based office. You feel incredibly worried when you don’t have your own room.’

### Business trips and virtual collaboration

The result in Table 7 reveals that, on average, more than 75% of the respondents made business trips often or sometimes before the COVID-19 pandemic, and among those that made business trips before, this dropped to almost 2% during the pandemic. The business travel thus almost disappeared. The lack of business travel seems, however, not to have affected the working situation gravely. On the question how meetings and other events that previously would have required travel were being carried out, presented in Table 8, an average of 3% of respondents answered that all or almost all meetings/errands had been cancelled and that 90% had managed to complete most of their business tasks using virtual collaboration. The result thus indicates that the business of the five public agencies was maintained. The Public Employment Agency was least affected, but that agency also had the lowest level of business trips for the beginning.

**Table 7:** Responses to the Questions ‘Did You Take Business Trips Before COVID-19?’ and ‘Do You Make Business Trips Now During COVID-19?’

Organisation	Business trips before COVID-19?			Business trips during COVID-19?	
	Yes, often	Yes, sometimes	No	Yes	No
Transport Administration	24%	66%	11%	2.8%	97.2%
Environmental Protection Agency	10%	68%	22%	0.0%	100.0%
Energy Agency	10%	64%	26%	0.0%	100.0%
Agency for Digital Government	19%	68%	13%	0.0%	100.0%
Public Employment Service	1%	42%	57%	1.6%	98.4%
All agencies	15%	62%	22%	1.8%	98.2%

*Note*: Separated by organisation and for all agencies

**Table 8:** Responses to the Question ‘How Do You Carry Out Your Meetings or Other Events for Which You Previously Travelled?’

Organisation	All or almost all meetings are cancelled	Most meetings are cancelled; for a few, virtual meetings are used	Some meetings are cancelled; for most, virtual meetings are used	Virtual meetings are used for all or almost all meetings
Transport Administration	2%	7%	26%	65%
Environmental Protection Agency	5%	10%	27%	58%
Energy Agency	3%	7%	26%	64%
Agency for Digital Government	7%	7%	17%	69%
Public Employment Service	1%	1%	12%	86%
All agencies	3%	6%	24%	67%

*Note*: Separated by organisation and for all agencies

In Table 9, the average rating regarding how well the virtual collaboration functioned is somewhat lower than the rating of telework, at 73.17 compared to 74.26. Analyses of the material also show that there is a correlation, though at a low degree, between the rated function of teleworking and that of virtual collaboration (Pearson correlation coefficient 0.60, *p* = 0.000). According to Table 10, younger respondents ranked how well virtual collaboration worked higher than older respondents, and women higher than men. The differences are not statistically significant though.

**Table 9:** Responses to the Question ‘How Well Do You Think Virtual Collaboration Has Worked for You in Your Work and for the Projects in Which You Participate (Rating 1–100)?’

Organisation	How well virtual collaboration works	
	Mean	Stdv
Transport Administration	75.17	16.22
Environmental Protection Agency	70.84	17.71
Energy Agency	71.01	17.23
Agency for Digital Government	69.65	19.90
Public Employment Service	74.24	17.01
All agencies	73.17	17.05
F-test (p-value)	2.57 (0.037)	

*Note*: Separated by organisation and for all agencies

**Table 10:** Responses to the Question ‘How Well Do You Think That Virtual Collaboration Has Worked for You in Your Work and for the Projects in Which You Participate (Rating 1-100)?’

Characteristics		How well virtual collaboration works			
		Female		Male	
		Mean	Stdv	Mean	Stdv
Age	20–29 years	72.35	19.47	70.42	14.77
	30–39 years	73.57	18.71	74.08	18.43
	40–49 years	74.82	16.69	72.85	16.21
	50–59 years	75.40	15.51	69.37	16.14
	60–69 years	71.85	17.61	73.00	17.80
	Average	74.29	16.97	72.21	16.97
F-test (p-value)		1.06 (0.363)		1.10 (0.300)	

*Note*: Separated by organisation and for all agencies

In the same way as for telework, the respondents were asked to reflect on virtual collaboration. Many comments from respondents were connected to virtual collaboration and the social dimension, e.g. ‘I have a fear of getting caught up in the mobile working methods, resulting in less social contact’ and ‘This adds greater importance and value to the social dimension once you meet physically in the office’. On the other hand, some respondents viewed the increased use of virtual collaboration as nothing extraordinary, e.g. ‘We were already doing a lot of virtual meetings, so the change won’t be too big, especially in international cooperation’. This type of comment corresponds well with the responses given to the question dealing with thoughts about virtual collaboration in the future (see Table 11). More than 90% of respondents believe themselves and colleagues will become much better at collaborating virtually; however, only 5% believe in a massive change in how they and their colleagues will work in the future.

**Table 11:** Responses to ‘How Do You Think the COVID-19 Pandemic Will Affect the Ways You and Your Colleagues Interact After the Pandemic is Over?’

Organisation	Not in any way, everything will go back to normal	We are getting better at virtual collaborating, but only minor changes	We are much better at collaborating digitally, major changes	Nothing will be the same again – the changes are here to stay!
Transport Administration	10%	62%	26%	2%
Environmental Protection Agency	2%	41%	50%	7%
Energy Agency	4%	52%	43%	1%
Agency for Digital Government	6%	35%	42%	16%
Public Employment Service	7%	39%	42%	11%
All agencies	7%	52%	36%	5%

*Note*: Separated by organisation and for all agencies

## Discussion

The results indicate, among other things, that there has been a massive change in commuting trips and business travel for the office workers in this study, despite Sweden’s relatively soft approach to tackle the COVID-19 pandemic. Few respondents believe in a drastic change in the way they and their colleagues work and collaborate in the future, but the majority believe that they will be much better at virtual collaboration. The results also show that the respondents were able to complete nearly all the meetings and tasks for which they had planned to travel, remotely and via virtual meetings. Before the pandemic, only a few thought that this was possible according to earlier studies on agencies involved in the REMM project.

Though this study targeted a similar working group, the result shows a variation in the adjustments made in meeting and travel behaviour and how well the respondents thought the adjustments worked. Looking at the adjustments in the number of commuting days during the pandemic (Fig. 1) it is important to keep in mind that the pandemic policy in Sweden is based on recommendations (e.g. work at home as much as possible and avoid public transport) and essentially it has been up to each agency, division and sometimes employee to decide on the adjustments made. Thus, though focusing on desk working staff we could expect a variation between the agencies studied due to variation in the type of work, number of external contacts, culture at the workplace, etc. This also suggest that we may have captured an even higher variation if including other employment sectors.

For reducing the environmental load from transport, teleworking and virtual meetings are possible measures [20]. If there is a wish to increase their use, it is worth noting that the results of this study indicate that excluding office presence entirely does not seem to be a viable option, due to the risk of missing out on essential communication as well as social interaction. Our results indicate that the best solution may be to travel to the office at least a few days a week.

As the design of this study prioritised an immediate setup, the study is subject to various pitfalls. In order to speed up the process, the sample of office workers was kept small. Further, as only the contact persons at the agencies had the contact information of all the employees who received the questionnaire, it has not been possible to analyse any skewness in the group of respondents. Comparing the gender share in the sample of this study with the gender share at the agencies, however, there is a general congruence. Beyond the sample size and representativeness, there are flaws connected to the responses to the survey. First, the responses are based on stated behaviour and not actual (revealed) behaviour; this issue with self-reported data is a common and well-recognised research problem. The results of this study should therefore be interpreted with this in mind. Second, there may be uncertainties as to whether the respondents remembered correctly how they behaved before the COVID-19 pandemic in questions concerning this time period. To minimise this problem, having the survey launched as soon as possible after the outbreak, was prioritised. There may also be uncertainties regarding the situation described in the survey questions as ‘now, during the COVID-19 pandemic’. During the peak of the pandemic in spring 2020, the situation, as well as the recommendations given to the population, changed rapidly; it was during these weeks that the survey was carried out. It is thus difficult to say exactly which point on the timeline that ‘during the COVID-19 pandemic’ represents. The results should therefore be interpreted as the general situation during the peak of the pandemic in Sweden during spring 2020. Despite these flaws, we are confident that the survey can give some guidance for the future.

Further, the sustainability potential of at least some of these behavioural changes remains large. According to the national travel survey (RVU Sweden, 2011–2014) [39], almost half of trips taken in Sweden are to work or school, or as business trips, and business travel accounts for 10% of the total number of passenger kilometres travelled per person per day. The effects in terms of reduced CO_2_ emissions as well as other negative effects related to transports, such as congestion and noise, are very promising indeed, though in need of support from policies to be realised. For these changes to become permanent, there is also a need for more radical changes in the culture around business travel. In this new approach to travel and meetings, revisions of existing workplace travel plans, and internal goals regarding business travel, may be seen as key factors.

## Conclusions

The results for all agencies together indicate that 86% of respondents changed their commuting trips under the COVID-19 pandemic, despite Sweden’s relatively liberal approach to restrictions. Regarding business travel, which is commonly seen as an intrinsic part of the business culture and thus difficult to change, only 3% of those who travelled from business before the pandemic continued to do so. For meetings, workshops, and other events for which the employees originally planned to travel, 88% of the respondents were able to complete all or the vast majority of meetings and other errands through the use of virtual meetings. Only 3% indicated that all or almost all meetings were cancelled.

These results indicate that the public agencies surveyed were well prepared and had a ‘backup collaboration solution’, at least technically, to make a rapid behavioural shift when travel was no longer an option. Though the Swedish government’s and Public Health Authority’s strong recommendations have led to the most dramatic reduction in work-related travel ever experienced in modern times, operations in Swedish agencies continued to function, as did worker communications and collaborations. These results indicate that there is great potential in a behavioural change in which digital tools provide an opportunity to influence if and how we commute and take business trips. The COVID-19 pandemic has shown that such tools can make the impossible possible.

## Data Availability

Follow-up surveys are planned with the project to capture long term effects of the COVID-19 pandemic. At the moment, the time plan is not settled due to difficulties predicting the development of the pandemic. While the project is ongoing, data will only be shared with the public agencies surveyed in the project. After project completion, aggregated data will be available.

## Footnotes

1 One of the public agencies participating in the study, the Agency for Digital Government, was established in 2018 and has been part of the REMM project since then.

2 Based on data from surveys in six public agencies involved in the REMM project and conducted in the period September 2017 – April 2019. In total 2164 employees responded to the six surveys. Data kindly provided upon request from the REMM project in June 2020.
